# The Carotenoid Diatoxanthin Modulates Inflammatory and Angiogenesis Pathways In Vitro in Prostate Cancer Cells

**DOI:** 10.3390/antiox12020359

**Published:** 2023-02-02

**Authors:** Clementina Sansone, Luigi Pistelli, Luana Calabrone, Angelo Del Mondo, Angelo Fontana, Marco Festa, Douglas M. Noonan, Adriana Albini, Christophe Brunet

**Affiliations:** 1 Stazione Zoologica Anton Dohrn, Sede Molosiglio Marina Acton, 80133 Napoli, Italy; 2 Laboratory of Vascular Biology and Angiogenesis, IRCCS MultiMedica, 20138 Milan, Italy; 3 Institute of Biomolecular Chemistry, Italian National Research Council (CNR), 80078 Pozzuoli, Italy; 4 Department of Biotechnology and Life Sciences, University of Insubria, 21100 Varese, Italy; 5 Unit of Molecular Pathology, Biochemistry and Immunology, IRCCS MultiMedica, 20138 Milan, Italy; 6 IRCSS European Institute of Oncology (IEO), 20141 Milan, Italy

**Keywords:** chemoprevention, oxidative stress-induced cell death, xanthophyll, angiogenesis, microalgae, diatoms, ferroptosis

## Abstract

Xanthophylls, a group of carotenoids, have attracted attention as human health benefit compounds thanks to their functionality and bioavailability. The great antioxidant and anti-inflammatory abilities of diatoxanthin (Dt), a photoprotective xanthophyll synthetized by diatoms, were recently documented. This study investigates the capacity of Dt to intercept prostate cancer progression in vitro on different human cell lines, exploring its role against cancer proliferation and angiogenesis. Our results highlighted the chemopreventive role of Dt already at low concentration (44.1 pM) and suggest that the Dt-induced cancer cell death occurred through oxidative stress mechanisms. This hypothesis was supported by variations on the expression of key genes and proteins. Oxidative stress cell deaths (e.g., ferroptosis) are recently described types of cell death that are closely related to the pathophysiological processes of many diseases, such as tumors. Nonetheless, the interest of Dt was further strengthened by its ability to inhibit angiogenesis. The results are discussed considering the actual progress and requirements in cancer therapy, notably for prostate cancer.

## 1. Introduction

Prostate cancer is one of the most common diagnosed malignancies and was the fifth cause of cancer-related deaths among men in 2020 in the world [1]. Therapeutic approaches include chemoprevention through lympho-angioprevention which can intercept disease progression by inhibiting or delaying metastasization [2]. Currently, chemopreventive synthetic agents are being evaluated in vitro, in vivo or in patients to prevent prostate cancer or its recidivism after remission and/or to reduce tumor mass [3]. Chemopreventive agents promote cancer cell death, activating regulated cell death (RCD) pathways and immunogenic cell deaths (ICD). Among non-apoptotic RCDs, oxidative stress-induced cell deaths such as ferroptosis were recently described and are often associated with a variety of human diseases, such as neurodegeneration, ischemia-reperfusion injury, and various cancers, including prostate cancer [4]. Oxidative stress regulation is mainly mediated by ion intake and the homeostatic maintenance of intracellular redox state through antioxidant molecules as well as glutathione [5]. In this context, ferroptosis is mediated by intracellular iron accumulation; this is a key feature for cancer cells which activate dysregulate iron addiction mechanisms leading to an iron intake-mediated high proliferation rate [6]. The inhibition of glutathione peroxidase 4 (GPX4) or solute carrier family 7 member 11 (SLC7A11) are key events in the onset of oxidative stress cell deaths such as ferroptosis [7], which is characterized by an increase of peroxidation of phospholipids enriched with PUFAs and reactive oxygen species (ROS) [8]. GPX4, through reduced glutathione (GSH), converts lipid hydroperoxides into lipid alcohols, thereby alleviating lipid peroxidation and inhibiting ferroptosis, while SLC7A11 is a transmembrane transporter that exchanges extracellular cystine for intracellular glutamate [9]. In neoplastic cells, oxidative stress might be an endogenous tumor suppressive mechanism downstream of tumor protein 53 (TP53), by targeting polyunsaturated fatty acids (PUFAs) metabolism through activation of acyl-coA synthetase long chain family member 4 (ACSL4) [4,5]. 

A recent study suggested that ferroptosis might be applied as therapy for prostate cancer [4]. Ferroptosis-inducers accepted by FDA to be used as drugs are generally from synthetic origin [10], while the fight against cancer requires new compounds, with an increased demand for natural-origin compounds. Natural chemopreventive compounds, referring to phytochemicals [3], generally promote the scavenging of intracellular free radicals and the development of RCD/ICD in neoplastic cells. A huge reservoir of secondary metabolites is represented by marine systems, known for their chemical diversity richness. Several marine compounds, to date mostly from animal origin, have been successfully tested and registered for therapy, e.g., trabectedin against sarcomas and ovarian cancer [11]. For bioethics, economic and eco-sustainable reasons, microbes are more suitable than invertebrates or in general multicellular organisms for their exploitation as a renewable resource. 

Photosynthetic aquatic microalgae are strongly attractive for biotechnological issues [12] such as biomedical purposes [13,14,15,16]. Among them, diatoms represent a valuable bio-platform to be used as a cell factory for sustainable production of biomolecules [17]. Diatoms are fast-growing microalgae [18] containing the xanthophyll diatoxanthin (Dt), which is one of the pillars of their ecological success [19,20] as they quickly and efficiently protect cells from environmental-induced damages (e.g., light [21,22,23]) and through modulating intracellular iron concentration variations [24]. The involvement of Dt in diatom iron metabolism [25] and its strong in vitro bioactive capacity even at low concentration as recently unveiled [26,27] led us to investigate its chemopreventive function through a potential role in the oxidative stress-induced cell death (e.g., ferroptosis) mechanism in human cells.

Our study explores Dt as a potential specific cell death inducer in two different prostate cancer cell lines at gene and protein levels. The capability of Dt to promote cell death through oxidative stress management alteration in cancer cells was investigated targeting the modulation of GSH pathway-related key factors and the potential modulation of the release of inflammatory mediators, in turn stimulating the innate immune system directly involved in the inhibition of the lymphangiogenesis pathway. Moreover, Dt induced inhibition of the vascular mimicry of prostate cancer cells, which refers to the ability of tumor cells to create their own vascular-like structures for nutrients and oxygen transport independently of the typical model of angiogenesis. The results of this study reveal that Dt interferes with endothelial cell morphogenesis on extracellular matrix in vitro, thereby strengthening the interest in Dt as a potential new natural chemopreventive compound.

## 2. Materials and Methods

### 2.1. The Human Cell Lines

Four human cell lines were used to investigate the effects of the xanthophyll Dt, namely prostate adenocarcinoma (PC3, ATCC, Manassas, VA, USA, CRL-1435), prostate carcinoma (DU145, ATCC, HTB-81), normal prostate epithelium (PNT2, ECACC, Salisbury, UK, 95012613) and endothelial cell line (HUVEC, ATCC, CRL-17309). PC3, DU145, and PNT2 cell lines were grown in RPMI 1640 medium supplemented with 10% (*v*/*v*) fetal bovine serum (FBS), 100 units mL^−1^ penicillin, 100 units mL^−1^ streptomycin and 2 mM of L-glutamine, in a 5% CO_2_ atmosphere at 37 °C. HUVEC cell line was grown in F-12K Medium (ATCC 30-2004) supplemented with 0.1 mg mL^−1^ of heparin solution (Sigma Aldrich, St.Louis, MO, USA, catalog #H3393) and with 10% (*v*/*v*) fetal bovine serum (FBS), and 1% of Endothelial Cell Growth Supplement (ECGS).

### 2.2. Diatoxanthin Cytotoxicity Assay 

To assess the potential cytotoxic effect of the pigment Dt, each human cell line was treated for 48 h with pigment-ethanol solution at three different concentrations—namely 4.41, 44.1 and 441 pM—and compared with untreated cells. At the end of the treatments, cells were incubated with 10 µL (5 mg mL^−1^) of 3-(4,5-dimethylthiazol-2-yl)-2,5-diphenyltetrazolium bromide (MTT) and incubated for 3 h at 37 °C with 5% CO_2_. The resulting formazan crystals, only produced by viable cells, were dissolved with 100 µL of isopropyl alcohol. The absorbance was recorded using a Microplate Reader: Infinite^®®^ M1000 PRO (TECAN, Männedorf, Switzerland) at a wavelength of 570 nm.

### 2.3. Morphogenesis on Matrigel

To assess the potential chemo-preventive activity we tested whether Dt would affect prostate cancer cells’ ability to form a network on Matrigel, resembling capillary formation (vascular mimicry). Vasculogenic mimicry assay was performed on DU145 prostate cancer cell line. A 96-well plate (from two to five wells per treatment) was pre-coated with Matrigel growth factor reduced (10 mg mL^−1^, 70 µL well^−1^; Corning, Corning, NY USA) that was allowed to polymerize for 30 min at 37 °C, 5% CO_2_. Then, 5 × 10^4^ DU145 cells were added to each well and treated with pigment or no treatment.

Angiogenesis on Matrigel was also tested. We looked at the capability of Dt to inhibit HUVEC cell capillary like formation on Matrigel upon growth factor stimulation. HUVEC cells (15 × 10^3^) were seeded into a 96 well plate, previously coated with 1 mg mL^−1^ of reduced growth factor Matrigel (Becton Dickinson, Franklin Lakes, NJ, USA). HUVEC cells were incubated for 24 h. Positive controls received 10% FBS EBM medium. The capillary network formation was determined using a Zeiss Microscope associated with a Nikon camera (Axio Observer A1, Carl Zeiss, Jena, Germany) and quantified with ImageJ software (U.S. National Institute of Health, Bethesda, MD, USA), using the Angiogenesis Analyzer tool.

### 2.4. RNA Extraction and Gene Expression Analysis

Cells (2 × 10^6^ cells) to be used for RNA extraction were seeded in 6-well plates (TPP Techno Plastic Products AG, Trasadingen, Switzerland) and kept overnight for attachment. For gene expression studies, cells were treated for 2 h with Dt at a concentration of 176.4 pM and compared with untreated cells. At the end of the treatment, cells were washed by adding cold 1X Phosphate Buffered Saline (Microgem, Naples, Italy). Cells were lysed directly in plates by adding 1 mL of TRIsure™ reagent (Cat. No. BIO-3803, Meridian Bioscience Inc., Cincinnati, OH, USA) and RNA was isolated according to manufacturer’s protocol. RNA concentration and purity were assessed using the NanoDrop 1000 Spectrophotometer (Thermo Fisher Scientific, Waltham, MA, USA). The reverse transcription reaction was carried out using RT2 First Strand Kit (Cat. No. 330404, Qiagen, Hilden, Germany). Quantitative Polymerase Chain Reaction (RT-qPCR) was performed using RT2 Profiler PCR Array Human Oxidative Stress Plus (384-well format, Cat. No. 330231, PAHS-065YA, Qiagen, Hilden, Germany). Plates were run on a ViiA 7 Real-Time PCR System (Thermo Fisher Scientific, Waltham, MA, USA). Standard Fast PCR Cycling protocol were run with 10 µL reaction volumes. Cycling conditions used were set up in three stages: the first stage corresponded to 50 °C for 2 min followed by 95 °C for 10 min; the second stage consisted in 40 cycles at 95 °C for 15 s and 60 °C for 1 min; the last stage (melt curve) corresponded to 95 °C for 15 s, then 60 °C for 1 min and 95 °C for 15 s. qPCR data (Ct-values) were analyzed with PCR Array Data Analysis Online Software (Qiagen Qiagen, Hilden, Germany). All values greater or lower than 2.0-expression ratios with respect to the controls were considered significant. Actin-beta (ACTB), beta-2-microglobulin (B2M), glyceraldehyde-3 phosphate dehydrogenase (GAPDH), hypoxanthine phosphoribosyl transferase 1 (HPRT1) and ribosomal protein large P0 (RPLP0) were selected as control genes for the real-time qPCR analysis.

### 2.5. Protein Expression Analysis

The production of SLC7A11, tumor necrosis factor receptor 1 (TNFR1) and sirtuin 2 (SIRT2) proteins was assessed performing a Western blot analysis on PNT2 and PC3 cell lines after 24 h treatment with Dt at a concentration of 44.1 pM. After incubation, the medium was removed while the cell lysates were prepared by scraping each well into 500 µL of RIPA Lysis and Extraction Buffer (Thermo Fisher Scientific, Waltham, MA, USA), supplemented with Halt™ Protease & Phosphatase Inhibitor Cocktail (Thermo Fisher Scientific, Waltham, MA, USA). The lysate was incubated on ice for 15 min and then clarified by centrifugation at 14,000× *g* for 20 min. Total protein concentration was determined according to the Bradford method using Bradford—Solution for Protein Determination (cat. No. A6932, Applichem, Darmstadt, Germany) with bovine serum albumin (BSA, cat. A2058, Sigma-Aldrich, St. Louis, MO, USA) as a standard. The protein extracts were stored at −20 °C until use. Before electrophoresis, protein samples were incubated at 95 °C for 5 min. Following, 10% SDS-PAGEs were stained with Coomassie Brilliant Blue R-250 Staining Solution (cat. No. 161-0436, Bio-Rad, Hercules, CA, USA) or blotted onto Trans-Blot Turbo Midi 0.2 µm Nitrocellulose membrane (cat. No. 170-4159, Bio-Rad, Hercules, CA, USA) using Trans-Blot Turbo Transfer System (cat. No. 170-4150, Bio-Rad, Hercules, CA, USA). Membranes were incubated for 1 h in blocking reagent (1X Tris Buffered Saline-TBS), with 0.1% Tween-20 with 5% *w*/*v* nonfat dry milk and incubated overnight at 4 °C with the primary antibodies diluted in 1X TBS, 0.1% Tween-20 with 5% BSA. TNFR1 (antibody 1:1000 diluted, ab19139, Abcam, Cambridge, UK), SLC7A11 (antibody 1:1000 diluted, cat. 126915, Cell Signaling Technology Inc., Danvers, MA, USA) and SIRT2 (antibody 1:1000 diluted, cat. 12650S, Cell Signaling Technology Inc., Danvers, MA, USA) proteins were investigated. Positive control was obtained by using an anti-β-Actin antibody (antibody 1:1000 diluted, cat. 4970, Cell Signaling Technology Inc., Danvers, MA, USA). After incubation, membranes were washed three times for 10 min each with 15 mL of TBS/Tween and then incubated with Goat Anti-Rabbit IgG H&L (Alexa Fluor^®®^ 488, antibody 1:500 diluted, ab150077, Abcam, Cambridge, UK) with gentle agitation for 1 h at room temperature. After incubation, membranes were washed three times for 5 min each with 15 mL of TBS/Tween. Blotted membranes were detected by visualizing proteins with ChemiDoc™ MP Imaging System (cat. No. 120-3154, Bio-Rad, Hercules, CA, USA). Densitometric analysis of immunopositive bands was performed using ImageLab software (Bio-Rad, Hercules, CA, USA).

### 2.6. Antibody Array

In order to investigate the regulation of the proteins involved in angiogenesis and inflammation processes, specific antibody arrays were performed using RayBiotech^®®^ C-Series Human Angiogenesis Array C1000 (code: AAH-ANG-1000, RayBiotech, Peachtree Corners, GA, USA) and RayBiotech^®®^ C-Series Human Inflammation Array C3 (code: AAH-INF-3, RayBiotech, Peachtree Corners, GA, USA). For this aim, PC3, DU145 and HUVEC cell lines (3.8 × 10^5^ cells) were seeded in 12-well plates (TPP Techno Plastic Products AG, Trasadingen, Switzerland) and kept overnight for attachment. For the detection of proteins, PC3 and DU145 cells were treated for 24 h at a concentration of Dt of 44.1 pM, while HUVEC cells were pre-treated for 1 h with tumor necrosis factor α (TNF-α) at a concentration of 10 ng mL^−1^ before the treatment with 44.1 pM of Dt for 24 h. 

After incubation, cell medium was collected. Protein concentration and purity were assessed using the NanoDrop 1000 Spectrophotometer (Thermo Fisher Scientific, Waltham, MA, USA). For each condition tested, 1 mL of sample was used to perform specific antibody array, according to manufacturer’s protocol. Blots were analyzed using ImageLab software (Bio-Rad, Hercules, CA, USA) and results shown in terms of relative expression.

### 2.7. Target Fishing and Docking Analysis

A preliminary target fishing was conducted to retrieve diatoxanthin potential interactors by using ACID online server [28]. Subsequently, the retrieved immune-system proteins in relation with molecular data were selected and used for binding affinity evaluation and pose. The 3D coordinates of the crystal structure of the proteins Solute Carrier Family 9 Member A1 (SLC25A12), Solute Carrier Family 25 Member 12 (SLC9A1), Fc Gamma Receptor Ia (FCGR1A) and Toll Like Receptor 4 (TLR4) (PDB id(s): 4P5X, 2YGG, 3RJD, and 4G8A, respectively) were retrieved from PDB-Protein Data Bank and taken as the receptor model in a flexible docking program. Proteins were optimized by UCSF Chimera 1.16 software [29] for removal of all heteroatoms and water molecules included in PDB files, while further polar hydrogen atoms were added to proteins to make the receptor molecules suitable for docking. Dt (CID 6440986) was prepared as ligand for docking following the same procedure. Gasteiger charges were added and maximum six numbers of active torsions were given to the lead compounds. Selected proteins were finally docked using molecular docking program AutoDock Vina 4.2.6 (La Jolla, CA, USA) [30]. 

## 3. Results

### 3.1. Dt as an Oxidative Stress-Related Cell Death Inducer in Prostate Carcinoma PC3 and DU145 Cells

**Figure 1:**
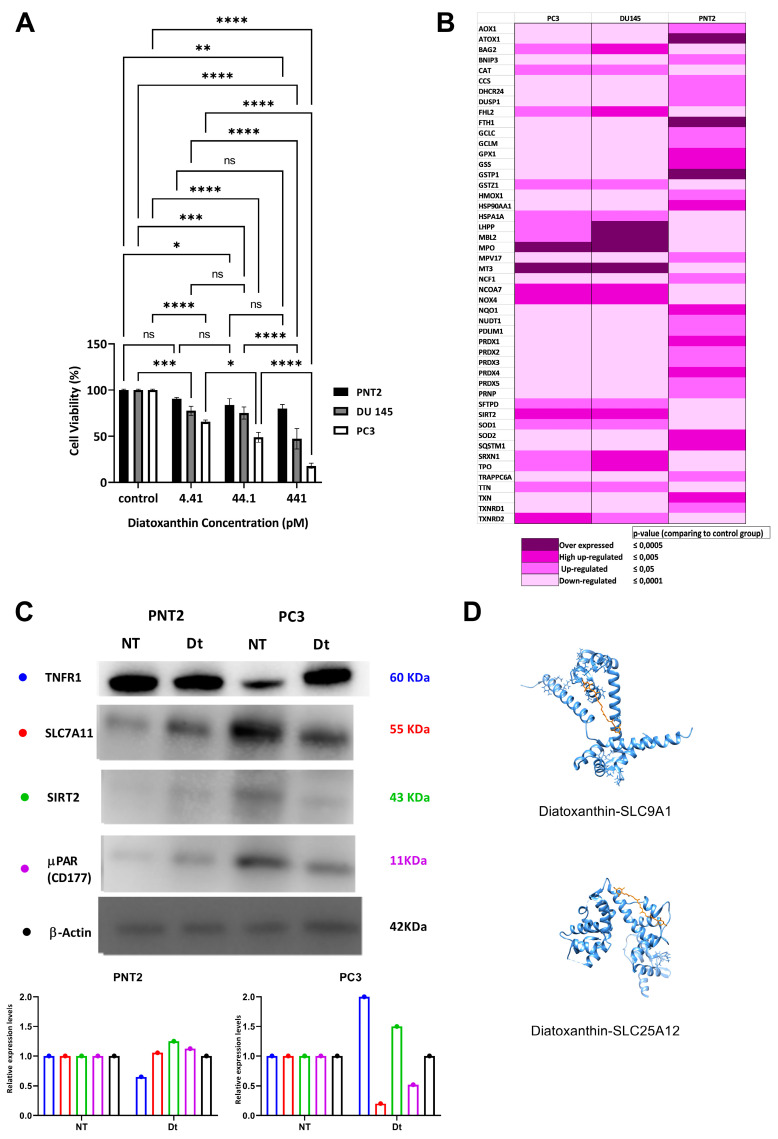
Effects of Dt on PNT2, DU145, and PC3 cells. (**A**) Percentage of viable human cells treated with 4.41, 44.1 or 441 pM of Dt. Histograms represent results obtained by MTT assay. (**B**) qPCR-array of oxidative stress-related genes after 2 h of incubation with Dt. (**C**) Western Blot analysis of TNFR1, SLC7A11, SIRT2, and μPAR (CD177) after 24 h of incubation with Dt on PNT2 and PC3 cells. (**D**) Molecular docking diagrams of Dt binding with SLC9A1 (up) and SLC25A12 (down). Asterisks indicate the statistically significant difference compared to the respective control (**** *p* ≤ 0.0001, *** *p* ≤ 0.001, ** *p* ≤ 0.01, * *p* ≤ 0.05; Sidak’s test), ns = non-significant different.

Dt did significantly inhibit growth of prostate carcinoma PC3 and DU145 cells in a dose-dependent way (Figure 1A). PC3 cells vitality was more affected by Dt than DU145 cells (Figure 1A), with an IC50 dose of 44.1 pM and 352.8 pM for PC3 and DU145 cells, respectively. The prostate PNT2 normal cells growth was very little affected by Dt (Figure 1A). Effects of Dt on the cancer PC3 and DU145 cell lines and normal PNT2 cell line led a modulation of the expression of many genes (Supplementary Materials: Table S1), generally opposed in cancer versus normal cells. Firstly, key genes involved in oxidative stress regulation (e.g., glutathione S-transferases (GSTs), peroxiredoxins (PRDXs)) were significantly downregulated in DU145 or PC3 cells while overexpressed in the counterpart normal prostate PNT2 cell line (Figure 1B). This was paralleled by a downregulation of the genes involved in cell protection against oxidative stress (e.g., antioxidant 1 copper chaperone (ATOX1), ferritin heavy chain 1 (FTH1), GPXs, PRDXs) in PC3 and DU145 while overexpressed in PNT2. Moreover, Dt induced an overexpression of the genes involved in the oxidative stress-mediated cell death (e.g., myeloperoxidase (MPO), metallothionein 3 (MT3), superoxide dismutases (SODs), 24-dehydrocholesterol reductase (DHCR24), SIRT2) in PC3 and DU145 cells, conversely to the observed downregulation of these genes in PNT2 cells (Figure 1B). In addition, genes encoding for functional products involved in autophagy regulation after iron-intake (i.e., sequestosome 1 (SQSTM1)) exhibited under-expression in cancer cells while they were up-regulated in PNT2 cells (Figure 1B).

Modulation of expression of the genes SIRT2, GPX4, SQSTM1, glutathione peroxidase 1 (GPX1), superoxide dismutases (SODs) and sulfiredoxin1 (SRXN1) in both cancer cell lines suggests the activation of ferroptosis-cell death mechanism. Indeed, up-regulation of SIRT2—increasing intracellular iron intake—simultaneously to the downregulation of SQSTM1—responsible for autophagic process for the homeostasis maintenance—might lead to intracellular iron toxicity, triggering a ferroptosis mechanism. The downregulation of the genes GPX1, GPX4 and SODs inhibits the development of cell responses to the iron accumulation-induced oxidative stress. The up regulation of the ferroptosis biomarker gene SRXN1 confirms the role of Dt in making prostate cancer cells (particularly DU145 cells) more sensitive to ferroptosis. In particular, the concentration of four known key-proteins (TNFR1, SLC7A11, SIRT2, µPAR: plasminogen activator, urokinase receptor (µPAR)) involved in the ferroptosis mechanism was compared in presence of Dt in the highly Dt-affected PC3 vs. normal PNT2 cells (Figure 1C). The two-fold increase of TNFR1 protein expression in PC3 compared to PNT2 cells confirmed the oxidative stress-driven induction in PC3 cells. The lowering of SLC7A11 protein expression in Dt-treated PC3 compared to PNT2 cells indicates lipid peroxidation (Figure 1C), one of the key steps of ferroptosis [31]. The 1.5-fold decrease level of SIRT2 in PC3, compared to PNT2 (Figure 1C) which causes iron ions to accumulate in the cell, occurred during ferroptosis. 

Since the SLC7A11 gene regulates glutamate/cysteine cell absorption for intracellular glutathione synthesis, its down-expression in PC3 treated with Dt (Figure 1C) indicated the blockage of cysteine intracellular intake and the inhibition of the up-stream glutathione biosynthetic pathway. The inhibition of glutathione synthesis, together with the high concentration of intracellular iron, represents the main cause for lipid oxidation. SLC7A11 gene, known as ferroptosis suppressor, is also associated with the two proteins, SLC25A12 and SLC9A1. The significant binding of Dt to SLC25A12 and SLC9A1 proteins was reliant on a hydrophobic interaction which is established among the α-helices (ΔE = −7.8 and −8 kcal mol^−1^, respectively), involving the active site of proteins, over-imposing standard ligand conformation (root-mean-square deviation of atomic positions—RMSD < 1; Figure 1D). 

The downregulation by Dt of µPAR, a ROS-dependent downstream factor which contributes to the malignant transformation and angiogenesis initiation of cells, in PC3 cells indicated a non-activation of angiogenesis and the conclusion of ferroptosis. 

### 3.2. Dt Modulation of Lymphangiogenic-Mediators Release 

**Figure 2:**
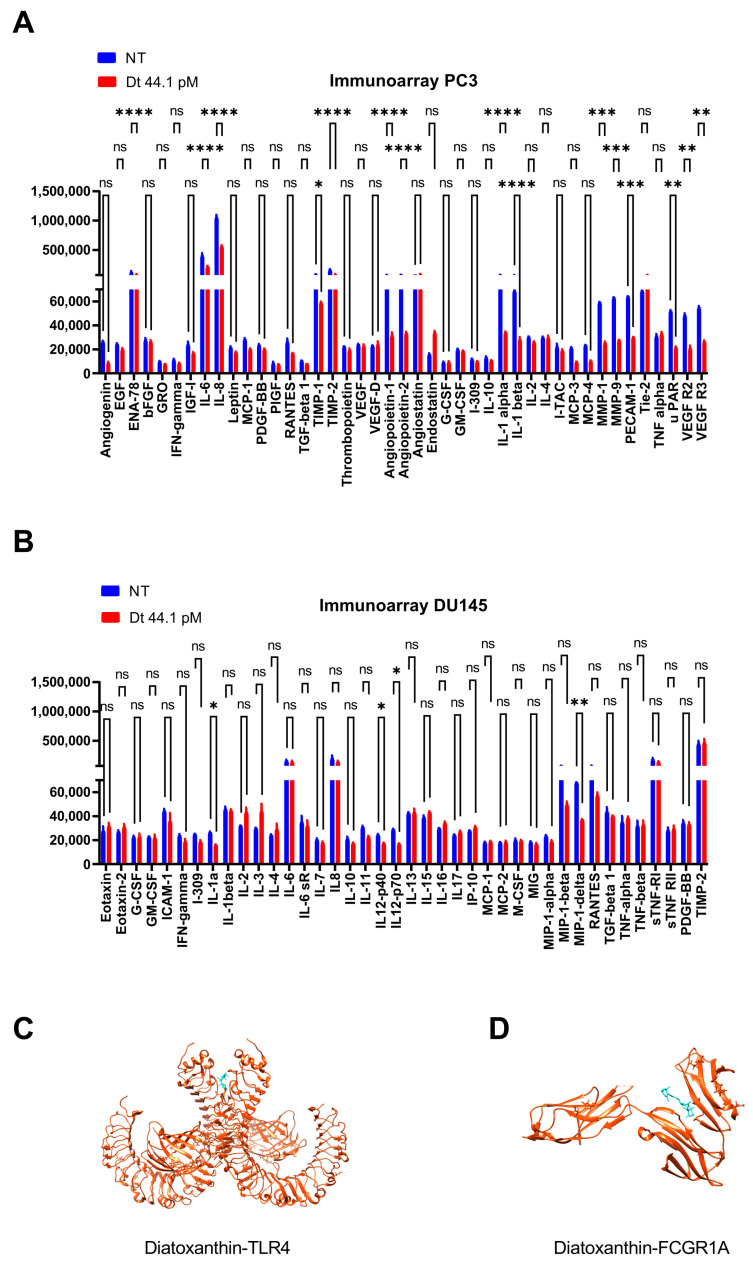
Angiogenesis inhibition in prostate cancer cell lines. (**A**) Human angiogenesis array analysis of the conditional medium from PC3 cells treated with Dt, and relative expression levels of the proangiogenesis cytokines. (**B**) Human inflammation array analysis of the conditional medium from DU145 cells treated with Dt and relative expression levels of the inflammation cytokines. (**C**,**D**) Molecular docking diagrams of Dt binding with TLR4 (**C**) and FCGR1A (**D**). Asterisks indicate the statistically significant difference compared to the respective control (**** *p* ≤ 0.0001, *** *p* ≤ 0.001, ** *p* ≤ 0.01, * *p* ≤ 0.05; Sidak’s test), ns = non-significant different.

The release of the 18 DAMP factors—angiogenin (ANG), interleukin 6 (IL-6), interleukin 8 (IL-8), metallopeptidase inhibitor (TIMP-2), angiopoietin 1 (ANGPT-1), angiopoietin 2 (ANGPT-2), C-C motif chemokine ligand 1 (I309—CCL1), interleukin 10 (IL-10), interleukin 1 α (IL-1α), interleukin 1 β (IL-1β), C-C motif chemokine ligand 7 (MCP-3—CCL7), C-C motif chemokine ligand 13 (MCP-4—CCL13), matrix metallopeptidase 1 (MMP-1), matrix metallopeptidase 9 (MMP-9), platelet and endothelial cell adhesion molecule 1 (PECAM-1), vascular endothelial growth factor receptor 2 (VEGFR2), vascular endothelial growth factor receptor 3 (VEGFR3), µPAR—was significantly lowered in the conditioned medium from PC3 cells treated with Dt compared to the untreated cancer PC3 cells (Figure 2A, Supplementary Materials: Figure S1A). µPAR release was strongly lowered (by ~65%, Figure 2A, Supplementary Materials: Figure S1A) in agreement with the Dt-induced decrease of µPAR expression level in Dt-treated PC3 cells compared to untreated cancer PC3 or to PNT2 cells (Figure 1C). The significant down-expression of ANGPT-1 and ANGPT-2 (Figure 2A, Supplementary Materials: Figure S1A) in Dt-treated PC3—compared to untreated cells—indicated an inhibition of the lymphangiogenesis pathway [32]. The consequent inhibition of the angiogenesis in Dt-treated PC3 cells was confirmed by the significant down-expression of MMP-1 and MMP-9 (Figure 2A, Supplementary Materials: Figure S1A). In addition, the decrease of MCP-3 and MCP-4 (Figure 2A, Supplementary Materials: Figure S1A) suggested a potential role of Dt in immunomodulation against tumor progression.

The Dt immunomodulation capacity was then explored in cancer DU145 cells through a human inflammation array analysis. DU145 cells represent the elective model for the study of the immunogenic cell death (ICD) induction via endoplasmic reticulum (ER) stress and ROS production for the exposure of different DAMPs [33].

Dt stimulated the expression of the macrophage inflammatory proteins (MIP-1-α, MIP-1-β and MIP-1-δ, Figure 2B, Supplementary Materials: Figure S1B) together with interleukins and chemokines (IL-6, IL-8, I309—CCL1, IL-10, IL-1α, IL-1β, MCP-3—CCL7, MCP-4—CCL13) responsible for the recruitment of immune cells that trigger the inflammatory storming against both the tumor instauration and disease progression in the presence of neoplastic cells. 

The ability of Dt to trigger immunomodulation in cancer cells was previously predicted by the significant binding affinity and pose between Dt and two selected proteins, FCGR1A, and TLR4 (Figure 2C,D). Dt binds FCGR1A through solely hydrophobic interaction which is established among the D1 and D2 domains (ΔE = −7.3 kcal mol^−1^), while Dt binds TLR4 thanks to the interaction between beta-barrel structures in proximity to dimerization domain (ΔE = −8.5 kcal mol^−1^). For both proteins, binding occurs super-imposed standard ligand conformation (RMSD < 1). 

### 3.3. Dt Inhibition of Vascular Mimicry and Capillary-like Morphogenesis

**Figure 3:**
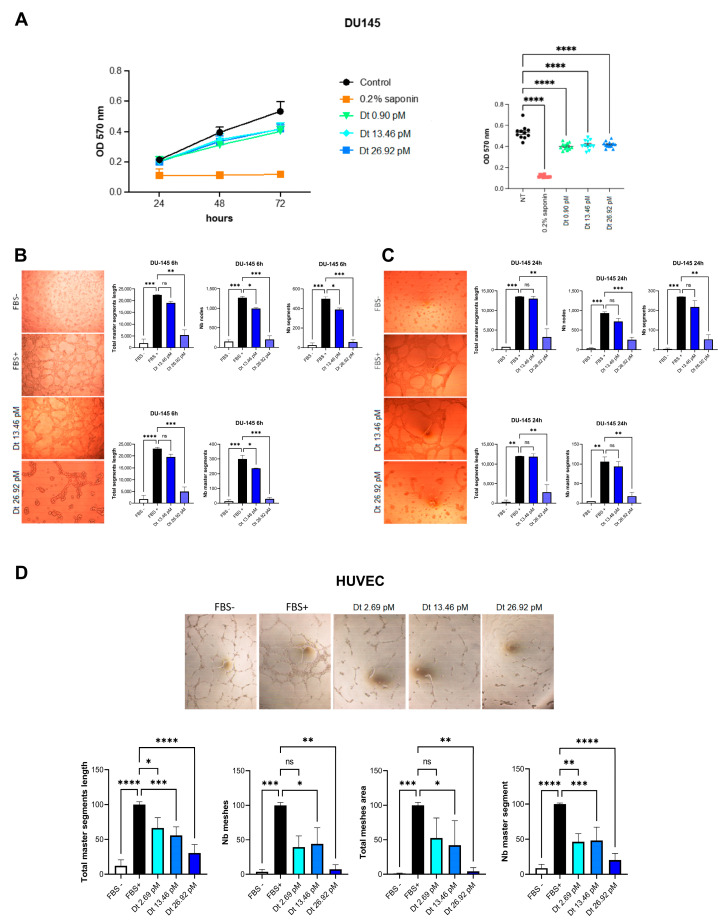
Assessment of vascular mimicry and angiogenesis inhibition on extracellular matrix. (**A**) Chemopreventive effects of Dt at three concentrations on DU145 cells compared to DU145 cells growth treated with saponin. (**B**,**C**) Capillary like morphogenesis of DU145 cells in absence or presence of Dt after 6 h (**B**) or 24 h (**C**) of treatment. (**D**) Capillary like morphogenesis of HUVEC cells in absence or presence of Dt after 24 h of treatment. Asterisks indicate the statistically significant difference compared to the respective control (**** *p* ≤ 0.0001, *** *p* ≤ 0.001, ** *p* ≤ 0.01, * *p* ≤ 0.05; Sidak’s test), ns = non-significant different.

DU145 cells treated with Dt showed that the concentrations of 13.46 and 26.92 pM slightly inhibited the cell viability after 48 and 72 h of treatment (Figure 3A). We therefore investigated the Dt role on the capillary mimicry inhibition at these two concentrations in DU145 cells over a time period less than 24 h (Figure 3B,C). Already after 6 h of treatment at 13.46 and 26.92 pM, total master segments length was reduced as well as Nb nodes and segments in comparison with FBS + cells (Figure 3B). This result was confirmed after 24 h (Figure 3C). The inhibition of network formation induced by Dt was verified at molecular level (Supplementary Materials: Figure S2A). The cytokines CXCR4, VEGF, IL-6 and TGF-β were all downregulated after treatment with 13.46 pM of Dt (Additional file 3: Figure S2A). In particular, the downregulation of the IL-6 gene expression determines the up-regulation of STAT3 transcription factor which is involved in the activation of IL6ST/gp130 signaling (Supplementary Materials: Figure S2A). In the Dt-treated HUVEC cells—the model cell line for in vitro angiogenesis exploration [34]—the antiangiogenic Dt activity was assessed (Figure 3D,E). Dt downregulated the gene expression encoding for proangiogenic factors (Supplementary Materials: Figure S2B) compared to cells stimulated before treatment, agreeing with the decrease of proangiogenic factors’ release (Supplementary Materials: Figure S2C). These results were also demonstrated with a capillary mimicry experiment performed on HUVEC cells, both with Dt treatment (Figure 3D,E) and with conditioned medium from prostatic carcinoma cells (Supplementary Materials: Figure S3), where Dt significantly inhibited network formation already after 6 h of treatment (Figure 3D) lasting for at least 24 h (Figure 3E).

## 4. Discussion

The carotenoid content and diversity of microalgae represents an under exploited reservoir of bioactive molecules. This is the case of the xanthophyll Dt [26,27], which in diatoms, is known to be highly efficient in protecting cells against high light damage [19,22]. Recent bioprospecting among 20 microalgal pigments demonstrated the in vitro great biological activity of Dt [26]. Based on previous investigations, this study advances a hypothesis on the ability of Dt to intercept cancer progression through the induction of a ferroptosis mechanism. Ferroptosis is an oxidative, iron dependent form of RCD [35], offering a new therapeutic opportunity for drug-resistant and metastatic cancers [36]. Our study verifies the hypothesis that Dt is a ferroptosis-inducer in prostate cancer cells. Depending on the cancer cell line, PC3 or DU 145 with different levels of aggressiveness, cells are more or less quickly affected by ferroptosis mechanisms. The higher the metastatic potential such as in the PC3 cell line [37] derived from human prostate cancer metastasis initiated from a bone metastasis of a grade IV, the quicker the activation of ferroptosis cell death. Conversely, ferroptosis cell death is little slower in DU145 cell line.

Dt activity displays a similar mechanism of action of the erastin [38]. It inhibits the cystine/glutamate antiporter system termed x−c, with the consequent cysteine depletion resulting in the lowering of the levels of GSH. Differently to Dt, in cancer cells treated with erastin and GPX4-deficient mouse cells, the accumulation of L-ROS, consumption of PUFAs and subsequent cell death can be prevented by treatment with small molecular antioxidants [39]. 

Ferroptosis belongs to ICD, releasing and activating DAMPs [15]. The capability of a molecule to trigger ferroptosis is mainly determined by its capacity to simultaneously inhibit different targets [40]. Among them is the cystine/glutamate antiporter system termed x−c which is a heterodimeric cell surface amino acid antiporter composed of the twelve-pass transmembrane transporter protein SLC7A11 (xCT) linked by a disulfide bridge to the single-pass transmembrane regulatory protein solute carrier family 3 member 2 (SLC3A2) [41]. The inhibition of cystine/glutamate antiporter induces an accumulation of intracellular iron [21]. The in-silico analysis carried out in this study suggests the straight binding between Dt and SLC9A1 and SLC25A12 predicting the inhibition of intracellular iron release through SLCs transporter. The dysregulation of iron homeostasis in prostate cancer cells increases basal metabolism, such as DNA synthesis, mitochondrial metabolism, leading to tumor proliferation, angiogenesis, and metastasis [42]. Iron accumulation does also cause oxidative stress, then inducing lipid peroxidation in prostate cancer lipid rich-cells, known to over-express ACSL4 [43]. Indeed, the activation of ferroptosis in cancer cells requires the overexpression of ACSL4 [4,5], of which the functional product enters into the PUFAs metabolism and is related to the depletion of glutathione reserve, a hallmark of cancer cells proliferation [44]. The deletion of ACSL4 gene confers resistance to ferroptosis in PC3 and DU145 cells [43], suggesting that ACSL4 gene expression level might be a proxy in predicting sensitivity to ferroptosis in prostate cancer cells. Whereas Dt exerts a greater antiproliferation role on PC3 than on DU145 cells (this study), in agreement with a higher ACSL4 gene relative expression in PC3 than DU145 cells [43], both cancer cell lines display activation of a similar ferroptosis pathway. The downregulation of SIRT2 protein by Dt further exacerbates iron accumulation and its intake through potential decrease of the FPN1 expression, which causes iron ions to accumulate in the cell, as they cannot be transported to the outside of the cell [45]. Dt also down-regulates all genes involved in Cys-dependent glutathione synthesis (e.g., glutathione synthetase (GSS), glutathione S-transferase Pi 1(GSTP1), glutathione S-transferase Zeta 1 (GSTZ1)) causing the depletion of glutathione reserves. The low level of intracellular glutathione does downregulate oxidative stress gene responses such as GPXs and PRDXs families (this study). Among GPXs, the role of GPX4 in initiating ferroptosis when downregulated is noteworthy [46]. The decreased level of intracellular glutathione was revealed through the Dt-inhibition of SLC7A11 together with SLC25A12 and SLC9A1 solute carriers (mitochondrial and cell membrane located, respectively) with the functions to import cystine for glutathione biosynthesis and antioxidant defense. Recent studies revealed that SLC7A11 overexpression promotes tumor growth partly in suppressing ferroptosis [47]. Our study reports that Dt-induced cancer cell death operates through a ferroptosis mechanism hypothesized by the results obtained on variations in expression of key genes and proteins. The tumor progression and metastasizing start from immune cells recruitment through lymphatic system invasion and involvement of angiogenic factors that promote the access in circulating blood, becoming a lymphangiogenesis [48]. 

In the present study, Dt increases the expression of vascular growth factors in PC3 and DU145 cancer cells as well as in HUVEC endothelial cells, indicating vasodilation and tissue permeability signaling to favor the diffusion of inflammatory mediators such as ILs, MIPs and TNF for the recruitment of immune cells. The immunomodulatory effect of Dt on PC3 and DU145 tumor cells is elicited by the inhibition of the release of the pro-lymphangiogenic interleukins and macrophage stimulating factors as well as interferon gamma (IFN-γ) in DU145 cells. IFN-γ is stimulated by the activation of TLR4, promoting tumor microenvironment immune surveillance and tumor progression [48]. The binding between Dt and TLR-4 revealed by in silico analysis might explain the downregulation of IFN-γ gene expression in DU145 cells and the inhibition of the release of pro-inflammatory interleukins and chemokines in PC3 and DU145 cells. In addition, TLR4 is known to induce VEGF expression in prostate tumors related to interaction with hypoxia inducible factor-1 α (HIF-1α); VEGF enhances the nuclear factor kappa-light-chain-enhancer of activated B cells (NF-κB) in the tumor microenvironment [49]. Indeed, HIF-1α gene expression is upregulated by Dt in the endothelial HUVEC cells. 

The high binding affinity between Dt and the FCGR1A, the latter representing one of the principal players in immune cell infiltration [50], highlights a potential immunomodulatory role of Dt in prostate cancer cells. This is a relevant finding since the FCGR1A receptor does interact with various immune cell marker genes in cancer cells, such as CD2 molecule (CD2), CD3 epsilon subunit of T-cell receptor complex (CD3E), CD86 molecule (CD86), CD163 molecule (CD163), V-set and immunoglobulin domain containing 4 (VSIG4), membrane spanning 4-domains A4A (MS4A4A), major histocompatibility complex, class II, DP β-1 (HLA-DP β1), major histocompatibility complex, class II, DR α (HLA-DR α), major histocompatibility complex, class II, DP α 1 (HLA-DP α-1), integrin subunit α-X (ITG α-X), and hepatitis A virus cellular receptor 2 (HAVCR2 also known as T-cell immunoglobulin and mucin-domain containing-3, TIM-3) in T cell, monocytes, M2 macrophages, dendritic cells, T-helper 1 lymphocytes, and causes T cell exhaustion [50]. 

Dt does also induce a decrease in inflammation and angiogenic factors in HUVEC cells previously inflamed with TNF-α. Indeed, the downregulation of the Kinase insert domain receptor (KDR, a type IV receptor tyrosine kinase) a cell-surface receptor for VEGFA, VEGFC and VEGFD, inhibits angiogenesis, partly via the activation of the ERK1/2 and Akt signal transduction pathways [51]. Indeed, the inhibition of VEGF/PI3K/mammalian targets of rapamycin (mTOR)/ERK signaling in HUVEC cells decreases angiogenesis, cancer growth, and metastasis [52]. The inhibition of pro-angiogenic signaling was also confirmed in a capillary mimicry experiment where both DU145 and HUVEC cells were not able to form an intercellular network after Dt treatment.

## 5. Conclusions

Tracking down chemotherapeutic agents from natural resources that can act specifically on cancer cells while avoiding side effects on somatic organs remains a daunting challenge. Photosynthetic microalgae represent a challenging eco-sustainable and renewable asset with potential human health benefit outcomes. Results from in vitro and in vivo experiments demonstrate the potential of the diatom xanthophyll Dt in fighting prostate cancer development. The Dt-activated role against the tumor is caused by the induction of oxidative stress-induced cell death mechanism (in cancer cells) as well as the inhibition of angiogenesis. Ferroptosis was the most likely suggested cell death mechanism induced by Dt as revealed by our data. This hypothetical mechanism needs to be confirmed by further studies. Furthermore, the properties of Dt may be used in combination with other chemotherapeutic agents to obtain a synergistic effect in preventing cancer progression [53]. This study paves the way for the development of Dt-based pharmacological or nutraceutical investigations. In this context, the *Odontella aurita* diatom, authorized as a food complement in the EU [54], might serve as a biomass vector for Dt assimilation in humans. The unearthed Dt bioactivity ([26,27], this study) may raise interest in the improvement of its synthesis in diatoms, for instance, by applying synthetic biology or genetic engineering tools [55]. 

## Data Availability

All of the data is contained within the article and the Supplementary Materials.

## Supplementary Materials

The following supporting information can be downloaded at: https://www.mdpi.com/article/10.3390/antiox12020359/s1, Table S1. Fold regulation values of genes obtained from PCR array genes expression analysis. Figure S1. (A) Human angiogenesis array blot of the conditional medium from PC3 cells treated with Dt, and relative expression levels of the proangiogenesis cytokines. (B) Human angiogenesis array blot of the conditional medium from DU145 cells treated with Dt, and relative expression levels of the proangiogenesis cytokines. Figure S2. (A) Proangiogenic cytokines expression levels by Real Time PCR on DU145 cells. (B) PCR array of proangiogenic factors-related genes in HUVEC cells stimulated with TNFα and treated with Dt. (C) Human angiogenesis array blot of the conditional medium from HUVEC cells treated with Dt after TNFα stimulation, and relative expression levels of the proangiogenesis cytokines. Figure S3. Effects of on capillary-like structure formation of HUVECs by PCa supernatants. Morphogenesis assay on HUVECs pre-treated with 50 μg/mL of CM collected from PCa cells following 24 h of treatment. Representative images of tubular structures with HUVEC exposed to (A) SFM, (B)CM from DU145 cells, (C) PC3 cells.

## Abbreviations

ACSL4	acyl-coA synthetase long chain family member 4
ACTB	actin-β
Akt	AKT serine/threonine kinases
ANG	angiogenin
ANGPT-1	angiopoietin 1
ANGPT-2	angiopoietin 2
ATOX1	antioxidant 1 copper chaperone
B2M	β-2-microglobulin
BSA	bovine serum albumin
CD2	CD2 molecule
CD3E	CD3 epsilon subunit of T-cell receptor complex
CD86	CD86 molecule
CD163	CD163 molecule
DAMPs	damage-associated molecular patterns
DC	dendritic cell(s)
DHCR24	24-dehydrocholesterol reductase
Dt	diatoxanthin
DU145	human prostate carcinoma cell line
ERK1/2	extracellular signal-regulated kinase 1/2
FBS	fetal bovine serum
FCGR1A	Fc gamma receptor 1a
FTH1	ferritin heavy chain 1
GAPDH	glyceraldehyde-3-Phosphate Dehydrogenase
GPX1	glutathione peroxidase 1
GPX4	glutathione peroxidase 4
GSH	glutathione
GSTP1	glutathione S-transferase Pi 1
GSS	glutathione synthetase
GSTs	glutathione S-transferases
GSTZ1	glutathione S-transferase Zeta 1
HIF1α	hypoxia inducible factor 1 subunit α
HLA-DPB1	major histocompatibility complex, class II, DP β 1
HLA-DRA	major histocompatibility complex, class II, DR α
HLA-DPA1	major histocompatibility complex, class II, DP α 1
HPRT1	hypoxanthin phosphoribosyl transferase 1
HUVEC	human umbilical vein endothelial cells
I309 (CCL1)	C-C motif chemokine ligand 1
ICD	immunogenic cell death
IFN-γ (IFNG)	interferon gamma
IL1α	interleukin 1-α
IL1β	interleukin 1-β
IL6	interleukin 6
IL8	interleukin 8
IL10	interleukin 10
ITGAX	integrin subunit α-X
KDR	kinase insert domain receptor
MCP3 (CCL7)	C-C motif chemokine ligand 7
MCP4 (CCL13)	C-C motif chemokine ligand 13
MIP-1-α (CCL3)	C-C motif chemokine ligand 3
MIP-1-β (CCL4)	C-C motif chemokine ligand 4
MIP-1-δ (CCL15)	C-C motif chemokine ligand 15
MMP-1	matrix metallopeptidase 1
MMP-9	matrix metallopeptidase 9
MPO	myeloperoxidase
MS4A4A	membrane spanning 4-domains A4A
MT3	metallothionein 3
MTOR	mechanistic target of rapamycin kinase
MTT	3-(4,5-dimethylthiazol-2-yl)-2,5-diphenyltetrazolium bromide
NF-κB	nuclear factor kappa-light-chain-enhancer of activated B cells
PC3	human prostate adenocarcinoma cell line
PECAM-1	platelet and endothelial cell adhesion molecule 1
PI3K	phosphoinositide 3-kinases
PNT2	human normal prostate epithelium cell line
PRDXs	peroxiredoxins
PUFAs	polyunsaturated fatty acids
RAG2	recombination activating 2
RCD	regulated cell death
RMSD	root-mean-square deviation of atomic positions
ROS	reactive oxygen species
RPLP0	ribosomal protein large P0
SIRT2	sirtuin 2
SLC3A2	solute carrier family 3 member 2
SLC7A11	solute carrier family 7 member 11
SLC9A1	solute carrier family 9 member A1
SLC25A12	solute carrier family 25 member 12
SODs	superoxide dismutases
SQSTM1	sequestosome 1
SRXN1	sulfiredoxin 1
SV40	Simian virus 40
TH1	human T helper 1 lymphocytes
TIM3 (HAVCR2)	hepatitis A virus cellular receptor 2
TIMP2	TIMP metallopeptidase inhibitor 2
TLR4	toll like receptor 4
TNF-α	tumor necrosis factor α
TNFR1	TNF receptor superfamily member 1A
TP53	tumor protein 53
VEGFA	vascular endothelial growth factor A
VEGFC	vascular endothelial growth factor C
VEGFD	vascular endothelial growth factor D
VEGFR2	vascular endothelial growth factor receptor 2
VEGFR3	vascular endothelial growth factor receptor 3
VSIG4	V-set and immunoglobulin domain containing 4
µPAR	plasminogen activator, urokinase receptor
