# Supplementary material for: The Carotenoid Diatoxanthin Modulates Inflammatory and Angiogenesis Pathways In Vitro in Prostate Cancer Cells
Source: Antioxidants (Basel). 2023 Feb 2;12(2):359. doi: 10.3390/antiox12020359 (PMC9952135; doi:10.3390/antiox12020359)
Supplement: Supplementary file 1 [file Figure S2.pdf › Figure S2.pdf]

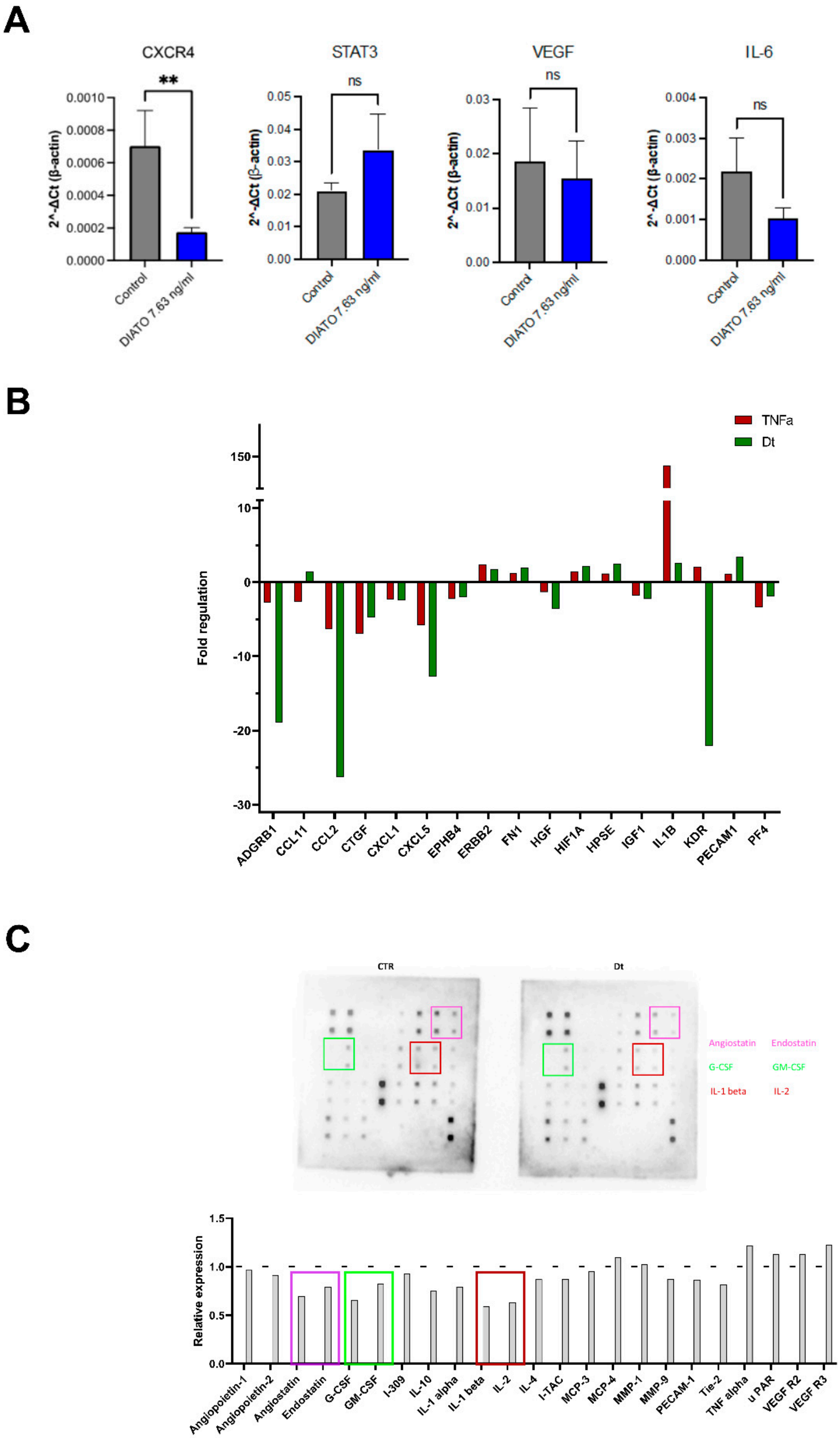

Supplementary Figure S2. (A) Proangiogenic cytokines expression levels by Real Time PCR on DU145 cells. (B) PCR array of proangiogenic factors-related genes in HUVEC cells stimulated with TNF and treated with Dt. (C) Human angiogenesis array blot of the conditional medium from HUVEC cells treated with Dt after TNF stimulation, and relative expression levels of the proangiogenesis cytokines.
