# Supplementary material for: The Carotenoid Diatoxanthin Modulates Inflammatory and Angiogenesis Pathways In Vitro in Prostate Cancer Cells
Source: Antioxidants (Basel). 2023 Feb 2;12(2):359. doi: 10.3390/antiox12020359 (PMC9952135; doi:10.3390/antiox12020359)
Supplement: Supplementary file 1 [file Figure S3.pdf › Figure S3.pdf]

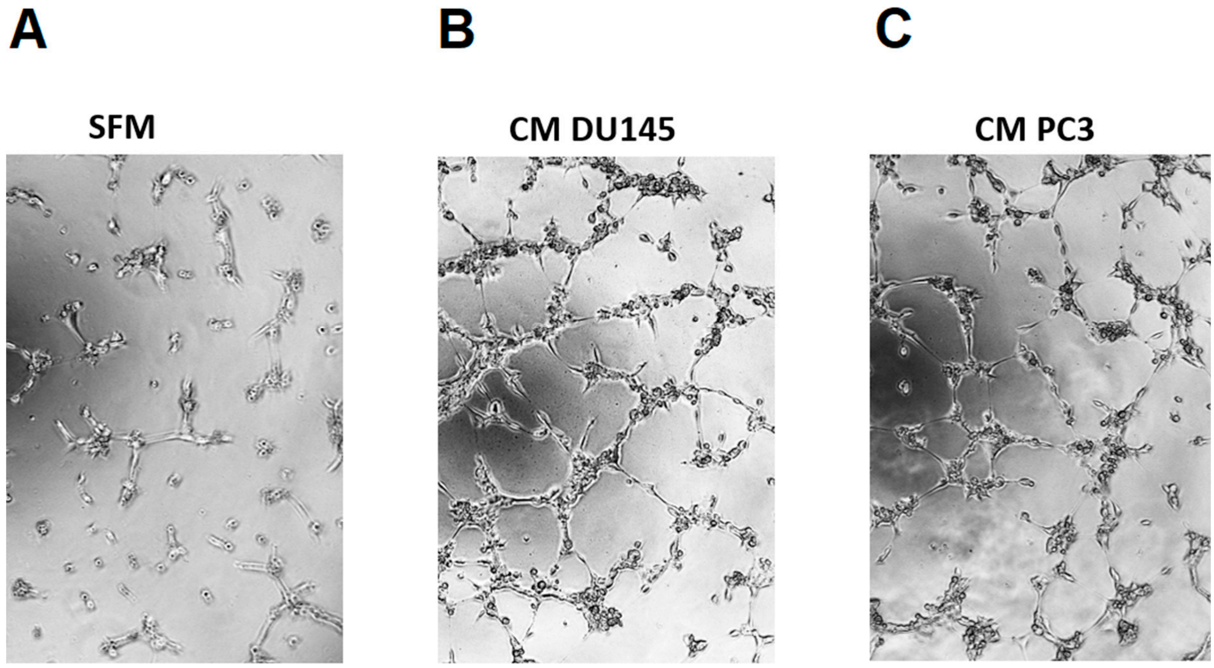


**Supplementary Figure S3.** Effects of on capillary-like structure formation of HUVECs by PCa supernatants. Morphogenesis assay on HUVECs pre-treated with 50 µg/mL of CM collected from PCa cells following 24 h of treatment. Representative images of tubular structures with HUVEC exposed to (A) SFM, (B)CM from DU145 cells, (C) PC3 cells.
