# Supplementary material for: The Carotenoid Diatoxanthin Modulates Inflammatory and Angiogenesis Pathways In Vitro in Prostate Cancer Cells
Source: Antioxidants (Basel). 2023 Feb 2;12(2):359. doi: 10.3390/antiox12020359 (PMC9952135; doi:10.3390/antiox12020359)
Supplement: Supplementary file 1 [file antioxidants-12-00359-s001.zip › Figure S1.pdf]

A

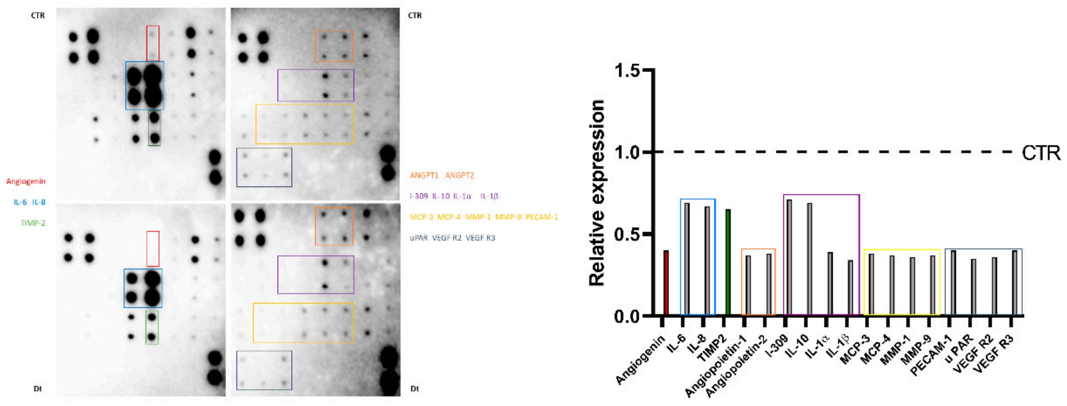

B

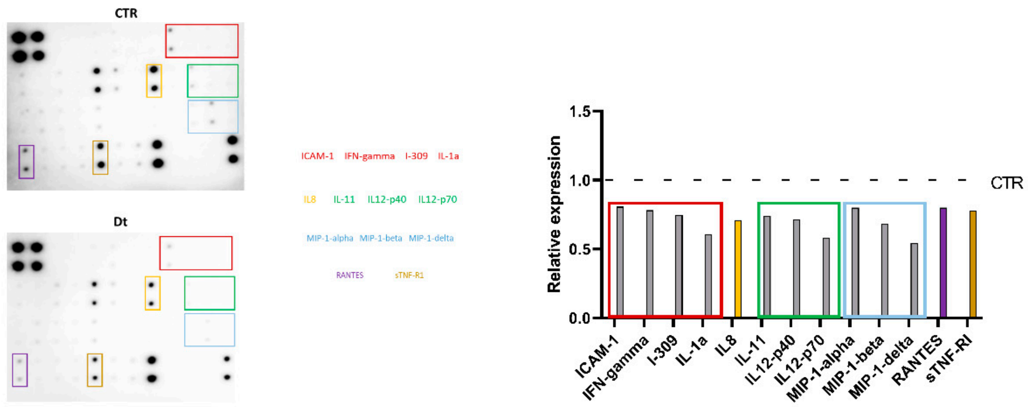

Supplementary Figure S1. (A) Human angiogenesis array blot of the conditional medium from PC3 cells treated with Dt, and relative expression levels of the proangiogenesis cytokines. (B) Human angiogenesis array blot of the conditional medium from DU145 cells treated with Dt, and relative expression levels of the proangiogenesis cytokines.
