# Supplementary material for: The Carotenoid Diatoxanthin Modulates Inflammatory and Angiogenesis Pathways In Vitro in Prostate Cancer Cells
Source: Antioxidants (Basel). 2023 Feb 2;12(2):359. doi: 10.3390/antiox12020359 (PMC9952135; doi:10.3390/antiox12020359)
Supplement: Supplementary file 1 [file antioxidants-12-00359-s001.zip › Table S1.pdf]

Table S1. Fold regulation values of genes obtained from PCR array genes expression analysis.

Gene Symbol	Gene Name	Fold Regulation		
		PC3	DU145	PNT2
ALB	Albumin	-5.90	1.01	-2.85
ALOX	Arachidonate 12-lipoxygenase	-1.17	5.79	-32.08
AOX	Aldehyde oxidase 1	-327.15	0.85	6.21
APOE	Apolipoprotein E	-5.90	1.61	-44.03
ATOX1	ATX1 antioxidant protein 1 homolog (yeast)	-33.60	0.80	1520.85
BNIP3	BCL2/adenovirus E1B 19kDa interacting protein3	-1.72	0.82	7.10
CAT	Catalase	12.54	1.11	-234.14
CCL5	Chemokine (C-C motif) ligand 5	-3.93	1.04	-2.37
CCS	Copper chaperone for superoxide dismutase	-40.60	1.42	12.08
CYBB	Cytochrome b-245, beta polypeptide	-3.04	1.78	-555.50
CYGB	Cytoglobin	-22.67	7.28	-7.30
DHCR24	24-dehydrocholesterol reductase	-49.20	3.91	66.64
DUOX1	Dual oxidase 1	-123.73	2.92	-1740.04
DUOX2	Dual oxidase 2	-10.80	5.75	-691.86
DUSP1	Dual specificity phosphatase 1	-1592.59	1.50	15.93
EPHX2	Epoxide hydrolase 2, cytoplasmic	-22.13	1.39	-367.91
EPX	Eosinophil peroxidase	-1.35	7.35	-82.53
FOXM1	Forkhead box M1	-514.00	1.64	1.91
FTH1	Ferritin, heavy polypeptide 1	-26499.59	0.96	3778.05
GCLC	Glutamate-cysteine ligase, catalytic subunit	-1.28	0.80	54.19
GPX1	Glutathione peroxidase 1	-54916.93	1.03	176.72
GPX2	Glutathione peroxidase 2 (gastrointestinal)	-5.55	1.41	-56.74
GPX3	Glutathione peroxidase 3 (plasma)	-2.20	2.37	-622.77
GPX4	Glutathione peroxidase 4 (phospholipid hydroperoxidase)	-16.40	1.41	1.70
GPX5	Glutathione peroxidase 5 (epididymal androgen-related protein)	-4.79	3.63	-10617.12
GSR	Glutathione reductase	-3.95	17.30	-42.72
GSS	Glutathione synthetase	-2217.57	1.02	137.20
GSTP1	Glutathione S-transferase pi 1	-279.77	0.93	1595.93
GSTZ1	Glutathione transferase zeta 1	12.54	0.96	-229.56
HSPA1A	Heat shock 70kDa protein 1A	59.05	3.56	-30.00
KRT1	Keratin 1	-5.90	0.91	-41.11
LPO	Lactoperoxidase	-5.90	0.02	-24.06
MB	Myoglobin	-3.65	1.04	-3.24
MBL2	Mannose-binding lectin (protein C) 2, soluble	78.98	0.02	-116.46
MPO	Myeloperoxidase	4429.08	0.77	-34270.58
MPV	MpV17 mitochondrial inner membrane protein	-2616.80	1.10	9.39
MSRA	Methionine sulfoxide reductase A	7.73	0.99	2.92
MT3	Metallothionein 3	29222.46	2.58	-29920.91
NCF1	Neutrophil cytosolic factor 1	-10.75	2.27	2.24
NCF2	Neutrophil cytosolic factor 2	-1.01	1.63	1.65
NOS2	Nitric oxide synthase 2, inducible	-5.67	0.57	-252.01

NOX4	NADPH oxidase 4	274.71	0.91	-30911.60
NOX5	NADPH oxidase, EF-hand calcium binding domain 5	1.11	2.21	-1.31
NUDT1	Nudix (nucleoside diphosphate linked moiety X)-type motif 1	-733.34	1.08	77.60
PDLIM1	PDZ and LIM domain 1	-442.55	0.83	18.36
PRDX1	Peroxiredoxin 1	-13.16	1.09	325.23
PRDX2	Peroxiredoxin 2	-34851.86	1.04	19.49
PRDX3	Peroxiredoxin 3	-2.28	0.69	35.82
PRDX4	Peroxiredoxin 4	-2.86	0.81	156.57
PRDX5	Peroxiredoxin 5	-62.60	1.04	2.31
PRDX6	Peroxiredoxin 6	-9.48	0.77	1.73
PRNP	Prion protein	-65.64	0.85	3.48
PTGS1	Prostaglandin-endoperoxide synthase 1 (prostaglandin G/H synthase and cyclooxygenase)	-1.42	1.29	-43.04
PTGS2	Prostaglandin-endoperoxide synthase 2 (prostaglandin G/H synthase and cyclooxygenase)	-7.28	2.32	-2.63
RNF7	Ring finger protein 7	1.62	0.58	1.55
VIMP	Selenoprotein S	-40.87	0.64	1.93
SEPP1	Selenoprotein P, plasma, 1	-2.13	0.52	-51.11
SFTPD	Surfactant protein D	79.11	1.24	-17.30
SIRT2	Sirtuin 2	608.29	4.23	-4412.90
SOD1	Superoxide dismutase 1, soluble	10.88	0.93	-2.13
SOD2	Superoxide dismutase 2, mitochondrial	-313.87	1.44	484.88
SOD3	Superoxide dismutase 3, extracellular	-48.50	2.70	-1.87
SQSTM1	Sequestosome 1	-376.14	1.23	150.80
SRXN1	Sulfiredoxin 1	7.52	2.36	-6.59
TPO	Thyroid peroxidase	2.77	125.15	-24.06
TTN	Titin	14.11	4.59	-2924.12
TXNRD2	Thioredoxin reductase 2	595.72	1.96	-580.28
UCP2	Uncoupling protein 2 (mitochondrial, proton carrier)	-5.01	1.45	-2.40
AKR1C2	Aldo-keto reductase family 1, member C2 (dihydrodiol dehydrogenase 2; bile acid binding protein; 3-alpha hydroxysteroid dehydrogenase, type III)	-1.27	1.12	-19.04
BAG2	BCL2-associated athanogene 2	17.33	0.68	-146.77
FHL2	Four and a half LIM domains 2	12.92	1.06	1.42
GCLM	Glutamate-cysteine ligase, modifier subunit	-30.28	0.88	4.25
GLA	Galactosidase, alpha	-66.78	0.88	-1.97
HMOX1	Heme oxygenase (decycling) 1	-68.47	1.07	5.04
HSP90AA1	Heat shock protein 90kDa alpha (cytosolic), class A member 1	-14312.07	0.84	932.67
LHPP	Phospholysine phosphohistidine inorganic pyrophosphate phosphatase	9.91	1.01	-6.82
NCOA7	Nuclear receptor coactivator 7	121.44	1.63	-338.55
NQO1	NAD(P)H dehydrogenase, quinone 1	-62.40	0.90	113.45

PTGR1	Prostaglandin reductase 1	2.67	0.97	5.73
SLC7A11	Solute carrier family 7 (anionic amino acid transporter light chain, xc- system), member 11	1.27	2.26	-3.24
SPINK1	Serine peptidase inhibitor, Kazal type 1	-14.55	1.11	-24.06
TRAPPC6A	Trafficking protein particle complex 6A	-25.79	0.84	8.15
TXN	Thioredoxin	-17.89	0.72	582.92
TXNRD1	Thioredoxin reductase 1	-5.90	0.87	69.26
